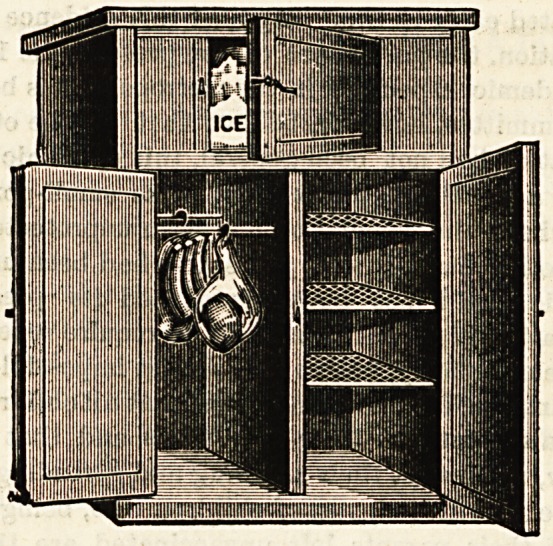# Modern Hospital and Institutional Fittings.—II

**Published:** 1903-11-07

**Authors:** 


### HOSPITAL ADMINISTRATION.

### CONSTRUCTION AND ECONOMICS.

### MODERN HOSPITAL AND INSTITUTIONAL FITTINGS.—II.

REFRIGERATION AND COLD STORAGE.

(*Continued from page* 73.)

Having seen how cold is produced, we must dwell on the methods of its transference to the cold storage chambers. This is brought about by one of three principal methods:—

1. *By Direct Expansion.*—Here the coils of pipes in which the gas expands (the expansor coils) are on the ceiling or walls of the chambers thus absorbing heat from the air directly. The expanded gas is kept circulating by the suction action of the compressors. This method is the simplest and most economical, and is particularly suitable where a low stationary temperature is required.

2. *Brine Circulation.*—In this case, as already mentioned, the expansor coils cool brine which is circulated by a special pump through insulated pipes to the cold storage rooms, which often are situated at a considerable distance. If there be more than one cold room each can have its temperature regulated to a certain level, and this, in such institutions as hospitals where the refrigerating effect is required for different purposes, needing varied degrees of temperature, proves its recommendation. We therefore find this method used more than any other.

3. *Air Blast.*—Here there are no pipes in the cold room, but above the room are the dry coils containing the expanded gas or coils over which brine is kept trickling, and over these air is blown by a powerful fan. The cold air rushes into the room to be cooled, from a delivery tube in the ceiling, and tends to sink, but getting gradually warmed, rises, and finally is sucked back through another shaft to be again blown over the coils and again cooled. This is the cheapest system to instal, but has the great disadvantage of drying the air too much, thus acting deleteriously on stored goods of a perishable nature.

The construction of the cold storage chamber is, of course, of great importance, in order to make sure that no heat can enter from outside sources. The shell of the building is nearly always constructed of match-boarding, in between the layers of which a special insulating material is placed. This consists commonly of 4 to 6 inches of charcoal, granulated cork, or slag wool. Atmospheric air is by far the best insulating medium we know of, and the above substances, holding air as they do, make use of its insulating properties. Specially prepared paper is also used in between the match-boarding to laminate the air for the same purpose, and as an inside coating, where it is often varnished to render it air-tight. For ordinary cold storage purposes we do not find much variation in construction.

Having somewhat briefly sketched out the various systems of mechanical refrigeration, it remains now to see how institutions avail themselves of this development of scientific progress and what systems are adapted to their special requirements. It will be well here to speak of non-mechanical refrigeration which many hospitals, clubs and hotels avail themselves of. The best known and most effective method is that known as the "Tasman Patent" (The "Tasman" Refrigerator Company, 40 Baltic Street, Golden Lane, E C.), of which the following is a description.

Storage is effected in a cabinet, made in different sizes according to requirements, and constructed of an outer and inner casing of matchboarding, in between the layers of which charcoal is placed to act as an insulating agent. For installing on yachts, etc., where much wear and tear is likely to be experienced, teak is used instead of matchboarding. Over the inner casing waterproof paper is fixed, and over this a lining of zinc. At the top of this chamber ice is placed on a shelf, which has on each side of it movable screens of galvanised iron with perforations in their upper part to prevent the full force of rising heated air striking the ice at once, thus economising ice consumption. On the back wall of the chamber is a small tank into which water from the melted ice may flow through sloping pipes from the ice shelf. As this water accumulates, it may be drawn off or special means may be taken to allow of an overflow. Special air-tight doors of course have to be fitted, and though no light is usually necessary, sometimes a window for this purpose is placed in the door; in which case two sheets of glass are used, the dead air in between acting efficiently as an insulating medium. This "patent" then depends for its effective working on the natural currents of cold air set up by the presence of the ice in the upper part of the chamber, and in practice its results are excellent.

Though such a method as this does not aspire to compete with mechanical refrigeration, yet for small institutions where a very low temperature is not required (average about 40° F.) it answers to all that is needed and has many advantages in its favour. The cost of working is only the cost of the ice (on an average 4 to 5 cwt. a week, costing about 2s. to 3s. per cwt. for an ordinary-sized chamber); there is nothing to go wrong and no skilled hands are required. It might be thought that the air would get too moist, but it is found that the natural circulation of the air is sufficient to obviate this, and lucifer matches left in the chamber all night will strike in the morning, which is a good test.

We find therefore that "The Tasman Refrigerator" is extensively used in many hospitals, restaurants, and hotels, where the cold storage requirements necessitate nothing more and where space forbids any apparatus on a larger scale.

*(To be concluded.)*

## Figures and Tables

**Figure f1:**